# Determination of the chemical and functional properties of yam bean (*Pachyrhizus erosus* (L.) Urban) flour for food systems

**DOI:** 10.1002/fsn3.574

**Published:** 2017-12-20

**Authors:** Evelyn S. Buckman, Ibok Oduro, Wisdom A. Plahar, Charles Tortoe

**Affiliations:** ^1^ Council for Scientific and Industrial Research – Food Research Institute Accra Ghana; ^2^ Department of Food Science and Technology Kwame Nkrumah University of Science and Technology Kumasi Ghana

**Keywords:** Chemical, Flour, Functional properties, *Pachyrhizus erosus*

## Abstract

Many plant species that are suitable for food across the world are neglected and underutilized. In order to increase their diversified food uses and thus help enhance food and nutrition security, we studied the chemical and functional properties of *Pachyrhizus erosus* (yam bean), which is a neglected and underutilized legume species. The chemical properties of flour produced from the yam bean include 5.8% moisture content, 5.7% crude fat, 6.2% crude fiber, and 85% available carbohydrate, indicating appropriate shelf‐stable flour, low fat, and abundant energy. The results also showed a reducing sugar content of 2.0% and 21.0% starch. Pasting temperature was 70.6°C with peak viscosity of 14.5 BU, which supports ease of cooking of the flour. The swelling power obtained was 752.9 g/100 g at 85°C with a solubility index of 54%. Water holding capacity (WHC) obtained for the flour was 363.88%, whereas swelling volume was 14.0 ml and makes the flour appropriate for the production of infant foods. The *P. erosus* flour therefore exhibits good functional and chemical properties that would make the flour quite suitable as a substitute for other flours in food systems.

## 1 INTRODUCTION

The present level of food insecurity in developing countries requires maximum research and development efforts to exploit and promote other food uses of all available neglected and underutilized food crops. Interestingly, most of these crops are indigenous and can easily be produced under the local climatic conditions. *Pachyrhizus erosus* is a legume plant belonging to the genus *Pachyrhizus*. The genus comprises five species (Sørensen, 1988). Three of these viz., *Pachyrhizus ahipa*, from Bolivia and Northern Argentina, *Pachyrhizus erosus* from the Semiarid tropics of Central America, Philippines, West Africa, and *Pachyrhizus tuberosus* from the tropical lowlands of the Adean mountain range are cultivated for their edible tuber and the remaining grow in the wild. The fresh yam bean tubers are rich in vitamin C and contain appreciable levels of neutraceuticals such as folates, riboflavins, pyridoxine, pantothenic acid, and thiamin, which when consumed in their right proportions qualify as functional foods. Studies by Hibon, Adegbola, Hell, and Thiele (2011) indicated that *P. erosus* tubers can be used to produce “*gari”* and flour*. Gari* is a roasted fermented dough prepared from cassava (*Manihot esculentu*), commonly prepared in West Africa. This is buttressed by studies by Zanklan, Ahouangonou, Becker, Pawelzik, and Grüneberg (2007), who reported the possibility of using *Pachyrhizus* root in the production of *gari* in Africa. Further studies by Forthsyth et al. (2002) on starch present in the tubers and seeds were done on six tuber accessions of *P. erosus*. Although yam bean has been demonstrated to have the potential to be integrated into the marginal, drought‐prone farming systems of sub‐Saharan Africa, where malnutrition is prevalent, it has never succeeded in becoming an established crop. The yam bean crop still remains an underutilized crop because of limited information on its physicochemical, functional, nutritional, and rheological properties for wider food uses. Information on these properties is necessary to enhance the utilization of yam bean in food systems. The yam bean can be processed into flour that can be used for the production of other food products like bread and pastries. Currently, research on alternative crops for flour production, substitution, and utilization is being proposed to provide alternative food forms at low cost in most sub‐Saharan countries. The objective of this work was to determine the chemical and functional properties of yam bean flour produced in Ghana.

## 2 MATERIALS AND METHODS

*Pachyrhizus erosus* tubers were obtained from Experimental Farms of the Council for Scientific and Industrial Research‐Crops Research Institute in Kumasi, Ghana.

### 2.1 Production of yam bean flour

The process for the development of the yam bean (*P. erosus*) flour was a modification of the method described by Dorporto, Mugridge, Garcia, and Vina (2011) for the production of *P. ahipa* flour. The yam bean tubers were washed thoroughly in water, peeled and then sliced to about 3.0 mm thickness using One‐Touch automatic deluxe vegetable Slicer (Model KC25, Daka Res. Inc., China) and weighed. The weighed samples were spread thinly on separate drying trays and dried in a mechanical dryer (Apex, Royce Ross Ltd) maintained at 55°C for 5.5 hr. The dried slices were weighed again and milled using a hammer mill (Jacobson Machine Works INC., Minneapolis, Minn, 55427, USA) to an average particle size of 250 μm. The resulting *P. erosus* flour sample was sealed in double laminated sealable polyethylene bags and stored in a freezer for further analysis (Badrie & Mellowes, 1993).

### 2.2 Proximate composition

Official methods of A.O.A.C. (2000) were used to determine moisture, ash, crude fat, total nitrogen, and crude fiber. Protein content was determined by multiplying % total nitrogen by a factor of 6.25. Available carbohydrate content was calculated by difference. Energy values were obtained using the Atwater factors 3.47, 8.37, and 4.00 for protein, fat, and carbohydrates, respectively (Eyeson & Ankrah, 1970).

### 2.3 Determination of sugars

Sugars were determined on clarified solutions before and after inversion at 68–70°C using the Lane and Eynon volumetric method as described by Pearson (1970). Thirty‐five grams of sample was weighed into a wide‐necked 250 ml volumetric flask. Extraction of water‐soluble matter was done by shaking the weighed sample with 150 ml of distilled water. The solution was clarified by the addition of 5 ml of zinc acetate followed by 5 ml of potassium ferrocynide solution. Content was made to the mark and filtered after 10 min. The specific sugars determined included total sugars, reducing sugars, and sucrose.

The sucrose content of the flour samples were determined in the absence of reducing sugars by inverting a portion of the test solution with acid followed by neutralization with alkali and titration (Pearson, 1970). The percent sugar was multiplied by 0.95 to get the percent sucrose content in the flour sample. For the determination of reducing sugars, a burette was filled with a quantity of the filtrate and this was used to titrate against 10 ml of equal volumes of Fehling's solutions A & B. Total sugars were determined by inversion. A 20 ml portion of the filtrate was quantitatively transferred into a 100 ml volumetric flask and distilled water added to produce a volume of 60 ml. Five milliliters of concentrated HCl was added and the flask swirled around and immersed in 68–70°C water bath for 10 min. The flask was cooled quickly and the solution neutralized with N NaOH, cooled and made up to 100 ml. This solution was used to titrate against 10 ml. equal volumes of Fehling's solution A & B as before.

### 2.4 Determination of starch content

The starch was determined using the Lintner's method (Pearson, 1970). About 0.5 g of the sample was titrated with 20 ml of water and 40 ml of hydrochloric acid added in small portions at a time. The mixture was then washed into a 200 ml flask with 12% (w/w) HCl, and 10 ml of 5% phosphotungstic acid added to precipitate proteins. The volume was made up to 200 ml with 12% more hydrochloric acid. The mixture was well shaken, filtered, and the optical rotation of the filtrate was measured in 200 mm tube. The mean specific rotation of *P. erosus* starch was taken as +185.7°.

### 2.5 Determination of pasting properties of P. erosus flour

The pasting properties of the flour were determined using the Brabender Viscoamylograph (No.802525, Duisburg, Germany) equipped with a 10 cm‐g sensitivity cartridge. Ten percent slurry of the flour sample was prepared with distilled water and the slurry was heated uniformly (1.5°C/min) from 25°C to 95°C, held at 95°C for 15 min, and cooled at the same rate to 50°C (Shuey & Tipples, 1982). The resulting amylograms provided pasting temperatures, peak viscosities, viscosity at 95°C, stability, cooking times, and setback viscosities.

### 2.6 Determination of swelling power, swelling volume, and solubility

The swelling power, swelling volume, and solubility of the flour were determined based on a modification of the method of Leach, McCowen, and Scotch (1959). One gram of the sample was transferred into a weighed graduated 50 ml centrifuge tube. Distilled water was added to give a total volume of 40 ml. The suspension was stirred uniformly with a stirrer avoiding excessive speed, in other not to cause fragmentation of the starch granules. The sample was heated at 85°C in a thermostatically regulated temperature bath (Grant instruments, England Ltd.) for 30 min with constant stirring. The tube was removed, wiped dry on the outside and cooled to room temperature. It was then centrifuged for 15 min at 560 g (Mistral 3000i, UK). The solubility was determined by evaporating the supernatant in a hot air oven (BS Gallenkamp, England) and the residue weighed. The swelling volume was obtained by directly reading the volume of the swollen sediment in the tube. The sediment paste was weighed. Determinations were done in triplicate.

### 2.7 Water‐binding capacity (WBC)

The WBC of the flour was determined in triplicate according to the method described by Aryee, Oduro, Ellis, and Afuakwa (2006). An aqueous suspension was made by dissolving 2 g of the sample in 40 ml of water. The suspension was agitated for 1 hr on Griffin Flask Shaker (Model hs501, digital, Janke 7 Kinkel GMBH &Co. KG) after which it was centrifuged for 10 min at 220 rpm with a centrifuge (Mistral 3000i, UK). The free water was decanted from wet starch and drained for 10 min. The WBC of the sample was then calculated as follows:

$$\text{Water binding capacity}=\frac{\text{Bound water}}{\text{Weight of sample}}\times 100$$

### 2.8 Statistical analysis

All determinations from the laboratory experiments were done in replicates. The statistical analyses were conducted using ANOVA. Significant statistical differences in samples were tested at *p *<* *.05, and least significant differences (LSD) method was used for the post hoc multi comparison test. All the analyses were done with Statistical Package for Social Scientists (16.0) software.

## 3 RESULTS AND DISCUSSION

### 3.1 Proximate composition

The proximate composition of the *P. erosus* flour is shown in Table 1. Flour with greater than 14% moisture is not stable at room temperature (Simsek, 2015). Organisms naturally present in the flour will start to grow at high moistures. The moisture content of *P. erosus* flour was about 5.8%**,** which is within the acceptable range for commercial flours. Comparing the root (83.7%) and flour (5.8%) moisture values, it is obvious that the oven drying of the samples was effective in causing a reduction in the moisture content of the flour to ensure long shelf‐life. The moisture content recorded for *P. erosus* flour is lower than that reported by Ukpabi (2010), who recorded moisture content of 9.4% for yam flour. Other research works on the proximate composition of cocoyam flour revealed a range of 5.01 to 15.20% moisture content.

**Table 1:** Proximate composition of *P. erosus*flour^a^

Parameter	As‐is Basis	DMB^b^
Moisture (%)	5.79 ± 0.04	–
Fat (%)	0.54 ± 0.01	0.57 ± 0.01
Ash (%)	2.14 ± 0.04	2.27 ± 0.04
Protein (%)	5.68 ± 0.06	6.03 ± 0.06
Crude fiber (%)	6.26 ± 0.01	6.64 ± 0.01
Carbohydrate by difference (%)	85.85 ± 0.15	91.12 ± 0.16
Reducing sugars (%)	11.34 ± 0.03	12.04 ± 0.03
Sucrose (%)	19.12 ± 0.01	20.29 ± 0.01
Total Sugars (%)	30.46 ± 0.40	32.33 ± 0.40
Starch (%)	21.00 ± 0.76	22.29 ± 0.81
Energy (kcal/100 g)	366.29 ± 0.14	388.79 ± 0.15

a Values are means ± standard deviation for triplicate determinations.

b DMB, Dry matter basis.

Crude fat content of the *P. erosus* flour was 0.54% (Table 1). When the crude fat content was expressed on dry‐weight basis, there was significant difference between the tuber and flour with respect to their fat content and this could be attributed to the processing procedure. Studies by McGill, Hard, Burt, and Gunstone (1974) showed decreases in fat contents of samples dried in the sun and attributed this to oxidation of fat during the period of drying. Dorporto et al. (2011) reported the crude fat content of 0.65% for *P. ahipa* on dry‐weight basis, which is similar to that obtained for *P. erosus* in this study. The difference could be due to varietal differences and/or differences in the geographical locations of their cultivation. The crude fat content obtained in this study is comparable to that reported by Padonou, Mestres, and Coffinago (2005) for the lipids content of cassava roots**.** The relatively low fat content recorded in this study makes *P. erosus* flour desirable as the risk of oxidation is reduced, thus preventing the development of off flavors resulting from rancidity. Also, the low fat content of the flour makes it a suitable substitute for health conscious individuals as well as over weights who want to reduce their caloric and fat intake.

Crude protein obtained for the *P. erosus* flour was 5.68% (Table 1). On dry‐weight basis, the protein content for the *P. erosus* tuber was about 9.6%. In studies conducted by Zanklan et al. (2007), the authors reported the protein content of *P. ahipa* (a pachyrhizus species*)* root flour as 9.0%, which is similar to the value obtained for the dried *P. erosus* tubers used in this study. This value is more than that of other root crops like cassava, sweet potato, and yam. Sabanis and Tzia (2009) also reported *P. ahipa* root flour protein content of 8.61%, lower than wheat flour (11.8%) but similar to corn (7.5%) and rice (7.0%). Similarly, *P. erosus* flour, due to its relatively high protein content and possibly its leguminous nature (with relatively high lysine and tryptophan content), can be composited with cereal flours and used as weaning foods for children to resolve nutritional deficiency problems. Probably, this can serve as a cost‐saving exercise since the current over‐reliance of soybean is becoming financially unbearable for most malnourished care givers.

Available carbohydrates, was 85.85% for the *P. erosus* flour (Table 1). This is not different from values reported in the literature (Adegunwa, Alamu, & Omitogun, 2011; Norman, Hoque, Haque, Pervin, & Karim, 2007; Ukpabi, 2010). The crude fiber content of the *P. erosus* flour was 6.26%, similar to that reported by Norman et al. (2007). Crude fiber measures the cellulose, hemicelluloses, and lignin contents of food.

Reducing sugar, total sugars, and sucrose contents of the *P. erosus* flour were 11.34%, 30.46%, and 19.12%, respectively (Table 1). The loss of moisture from the tubers led to an increase in total dry solids of the *P. erosus* flour, which had high concentration of total and reducing sugar content. Interestingly, processing significantly increased the content of sugars due to sacharification. High reducing sugars content in the tuber can cause darkening as a consequence of Maillard reaction between reducing sugars and amino acids, which is undesirable. Low content of reducing sugars is preferred as they result in lighter color of desirable quality (Abong, Okothl, Karuril, Kabira, & Mathooko, 2009). However, incorporation of *P. erosus* flour in the formulation of infant foods has an advantage of already having enough sugar thus the need for external or commercial sugar addition becomes unnecessary.

The starch content of the *P. erosus* flour was 21.0% (Table 1). On dry‐weight basis, these values were calculated to be 31.8% and 22.3% for the root and flour, respectively. Probably, the processing treatments might have caused the reduction in the starch content of the flour through possible hydrolysis. However, the starch content of the samples used in this study was less than that reported in earlier studies (Adegunwa et al., 2011; Elevina, Antonieta, Carmen, & Romel, 2012; Norman et al., 2007).

### 3.2 Pasting properties

**Figure 1:**
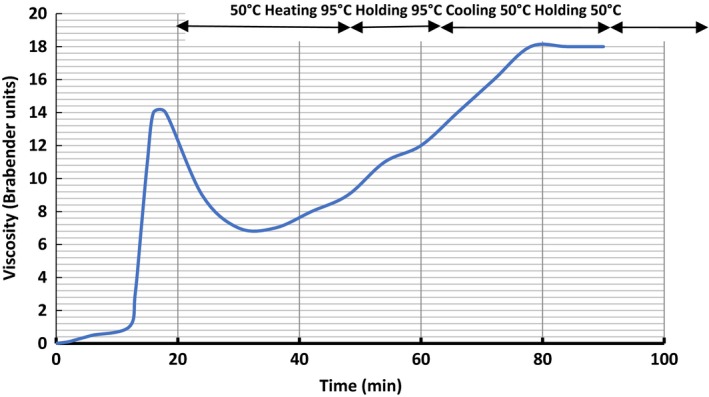
Amylograph pasting characteristics of standardized *P. erosus* flour

The pasting profile of *P. erosus* flour is shown in Table 2 and Figure 1. The *P. erosus* flour produced a pasting temperature of 70.6°C which is lower than that for sweet potato or taro flour (Aprianita, 2010). Melo, Silva, Krieger, and Stamford (2003) reported that the gelatinization temperature of yam bean starch paste ranges from 53 to 63°C, which is similar to that of cassava. Generally, pasting temperature of tuber starches is lower than cereal starches. The pasting temperature is one of the pasting properties which provide an indication of the minimum temperature required for sample cooking, energy cost involved, and stability of other components (Shimelis, Meaza, & Rakshit, 2006). Results indicated that *P. erosus* flour will cook faster with less energy consumed, thereby saving cost and cooking time because of its lower pasting temperature. Ease of cooking, which was estimated as the time from gelatinization to peak viscosity was 2.0 min for the *P. erosus* flour, which was less than 14 min reported for some legume flours by Plahar, Annan, and Nti (1997).

**Table 2:** : Pasting properties of *P. erosus* flour

Pasting characteristics	*P. erosus* flour
Pasting temperature (°C)	70.55 ± 0.07
Peak viscosity (BU)	14.50 ± 0.71
Peak time (min)	15.92 ± 0.35
Viscosity after Holding (BU)	7.00 ± 1.41
Final viscosity (BU)	16.50 ± 2.12
Break Down (BU)	7.50 ± 2.12
Set back (from peak)	2.00 ± 0.71
Setback (from holding at 95°C)	9.50 ± 2.83

The peak viscosity, which is the maximum viscosity developed during or soon after the heating was 14.5 BU at a peak time of 16 min. This is about half the peak viscosity value recorded for sweet potato (34.52 BU) and only a small fraction of the value for taro flour (267.9 BU). Oguntunde (1987) reported that the associative bonding of the amylose fraction is responsible for the structure and pasting behavior of flour‐starch granule. The viscosity or consistency of a cooked starch paste simply reflects the resistance to stirring of the swollen mass gel particles.

The breakdown viscosity of the *P. erosus* flour during prolonged cooking at 95°C was 7.5 BU. This is the difference between the peak viscosity and the viscosity after holding for 15 min at 95°C, an indication of the paste stability. Adebowale, Sanni, and Awonorin (2005) reported that the higher the breakdown in viscosity, the lower the ability of the sample to withstand heating and shear stress during cooking. Low starch paste stability is therefore commonly accompanied with high value of breakdown in viscosity Shimelis et al., 2006). Clearly, *P. erosus* flour was able to withstand more heating and shear stress than taro flour (15.3 BU) and sweet potato flour (23.40 BU). The final viscosity was 16.50 BU, producing a setback viscosity of 2.00 BU from peak (and 9.50 BU from holding at 95°C). Final viscosity indicates the ability of starch to form various paste or gel after cooling (Shimelis et al., 2006). Sanni, Ikuomola, and Sanni (2001) reported that lower setback viscosity during the cooling of gari indicates higher resistance to retrogradation. Therefore, *P. erosus* flour exhibited higher resistance to retrogradation due to its low set back value.

### 3.3 Functionality of *P. erosus* flour

The swelling power is the measure of the ability of starch to imbibe water and swell, and reflects the extent of associative forces within the granules (Moorthy & Ramanujam, 1986). Higher swelling index indicates higher associative forces. The swelling power obtained for *P. erosus* flour was 752.9 g/100 g at 85°C with a solubility index of 54% (Table 3). According to Dorporto et al. (2011), swelling power is temperature dependent and is accompanied by solubilization of starch granule constituents. In earlier studies with *P. ahipa* flour, Dorporto et al. (2011) reported swelling power values of 800 g/100 g sample. Swinkles (1985) reported that yam bean starch has a lower swelling power than cassava starch. The high swelling power of potato would be due, among others, to the high phosphate content because the negative charges allowed easier water entrance into the granules. High solubility was associated with high content of amylose, which leaches out easily during the swelling process (Sanni et al., 2001). Furthermore, high amylose content was linked with low swelling power due to greater reinforcement of the amylose molecules (Hoover, 2001). Highly associated starch granules with an extensive and strongly bonded structure also exhibit resistance to swelling (Leach et al., 1959). According to Richard, Asaoka, and Blanshard (1991) and Singh, Singh, Kaur, Sodhi, and Gill (2003), pasting viscosities are positively correlated. Thus the higher the swelling power of the sample, the higher the pasting viscosities. The relatively higher swelling power obtained in this study gives an indication of high amylose content likely to be present in the starch granules of the *P. erosus* flour.

**Table 3:** Swelling power, solubility, water holding capacity, and swelling volume of *P. erosus* flour

Functional property	Mean value
Swelling power at 85°C (g hydrated flour/100 g dry flour)	752.9
Solubility (%)	54
Water holding capacity (%)	368.88
Swelling volume(ml)	14

Water holding capacity (WHC) is an important parameter to be considered in the preparation of mashed snack foods and extruded foods as well as baked products. Water holding capacity (WHC) obtained for the flour sample was 363.88% (Table 3). The WHC is useful in explaining and predicting the behavior of food products (Wooton & Bamunuarachahi, 1978). In their studies on *P. ahipa*, Dorporto et al. (2011) reported WHC of 191% and 132% for flours produced by slicing and grating the tubers, respectively. Interestingly, lower WHC was reported by Osundahunsi, Fagbemi, Kesselman, and Shimoni (2003) for red sweetpotato flour (24%) and white sweetpotato flour (26%). The high values obtained in this study was possibly due to the relatively high protein content of the *P. erosus* root, which is similar to that reported by Ikegwu, Nwobasi, Odoli, and Oledinma (2009). High water holding capacity will give rise to high swelling power, and high peak viscosity. Root and tuber flours with high level of water absorption capacity will therefore be useful in meeting the needs for root and tuber incorporation into wheat flour for the bakery industry. Increase in WHC in food systems enables bakers to manipulate the functional properties of dough in bakery products (Achinewhu & Orafun, 2000). The higher WHC recorded for *P. erosus* flour makes it suitable for the production of mashed foods as it ensures product cohesiveness and also increase the unit yield of the product.

The swelling volume recorded for *P. erosus* flour was 14.0 ml (Table 3). Swelling volume (volume of gel) is used to measure flour or starch swelling properties. It is the volume of the un‐dissolved sediment obtained after centrifugation. The flour swelling volume test measures the cumulative effects of starch quality, specifically amylose/amylopectin ratio as reflected by the volume of gel produced when flour is heated with an excess of water.

## 4 CONCLUSION

The low moisture content of the *P. erosus* flour is appropriate to guarantee a stable shelf‐life of the product. Its low fat content also reduces the risk of oxidation and prevents off‐flavor development resulting from rancidity. The low fat content of this flour is advantageous as a substitute for health food particularly for over‐weight individuals. The high protein content of *P. erosus* flour makes it quite suitable for weaning foods for children, and the high sugar content and lower pasting as well as the high water holding capacity makes it better flour in the production of infant supplement foods. These desirable attributes of the *P. erosus* flour make it suitable for partial or whole substitution of traditional flours for the production of different flour‐based foods.

## CONFLICT OF INTEREST

None declared.

## References

[fsn3574-bib-0001] Abong, G. O. , Okothl, M. W. , Karuril, E. G. , Kabira, J. N. , & Mathooko, F. M. (2009). Levels of reducing sugars in eight Kenyan potato cultivars as indicated by stage of maturity and storage conditions. Journal of Animal and Plant Sciences, 2(2), 76–84.

[fsn3574-bib-0002] Achinewhu, S. C. , & Orafun, J. T. (2000). Fufu yield of some improved cassava cultivars and their physicochemical and sensory properties. Nigerian Food Journal, 17(18), 226–230.

[fsn3574-bib-0003] Adebowale, A. A. , Sanni, L. O. , & Awonorin, S. O. (2005). Effect of texture modifiers on the physicochemical and sensory properties of dried fufu. Food Science and Technology International, 11(5), 373–382. https://doi.org/10.1177/1082013205058531

[fsn3574-bib-0004] Adegunwa, M. O. , Alamu, E. O. , & Omitogun, L. A. (2011). Effect of processing on nutritional content of yam and cocoyam tubers. Journal of Applied Biosciences, 46, 3086–3092.

[fsn3574-bib-0005] AOAC . 47.021 ‐ 47.027 (2000). Official Methods of Analysis. 14th edition Association of Official Analytical Chemists. AOAC, St. Paul, MN.

[fsn3574-bib-0006] Aprianita, A. (2010). Assessment of underutilized starchy roots and tubers for their applications in the food industry. A thesis submitted in fulfillment of the requirements of the degree of Master of Science. School of Biomedical and Health Sciences, Victoria University, Werribee Campus, Victoria, Australia. pp. 135.

[fsn3574-bib-0007] Aryee, F. N. A. , Oduro, I. , Ellis, W. O. , & Afuakwa, J. J. (2006). The physicochemical properties of flour samples from the roots of 31 varieties of cassava. Food Control, 17, 916–922. https://doi.org/10.1016/j.foodcont.2005.06.013

[fsn3574-bib-0008] Badrie, N. , & Mellowes, W. A. (1993). Extrusion processing of cassava: Formulation of snack. In cassava flour and starch, Progress in Research and development. Chemistry, 75, 67–77.

[fsn3574-bib-0009] Dorporto, M. C. , Mugridge, A. , Garcia, M. A. , & Vina, S. Z. (2011). Pachyrhizus ahipa (Wedd.) Parodi roots and flour: Biochemical and functional characteristics. Food Chemistry, 126, 1670–1678. https://doi.org/10.1016/j.foodchem.2010.12.053 25213943 10.1016/j.foodchem.2010.12.053

[fsn3574-bib-0010] Elevina, E. P. , Antonieta, M. , Carmen, L. D. , & Romel, G. (2012). Roots, Tuber, Grains and Banana; Flour and Starches utilization in the development of food for convention, celaic and Phenylketonuric consumers. Journal of Processing and Technology, 4, 211.

[fsn3574-bib-0011] Eyeson, K. K. , & Ankrah, E. K . (1970). Composition of foods commonly used in Ghana. Accra, Ghana: Food Research Institute, Council for Scientific and Industrial Research.

[fsn3574-bib-0012] Forthsyth, J. L. , Ring, S. G. , Noel, T. R. , Parker, R. , Cairns, P. , Findlay, K. , & Peter, R. S . (2002). Characterization of starch from tuber of yam bean (*Pachyrhizus ahipa*). Journal of Agric. and Food Chem, 50(2), 361–367. https://doi.org/10.1021/jf0108922 11782208 10.1021/jf0108922

[fsn3574-bib-0013] Hibon, A. , Adegbola, P. Y. , Hell, K. , & Thiele, G . (2011). Contraintes et Opportunités pour l'Introduction de Nouveaux Produitssur les Marchés Locaux des Racines et Tubercules au Bénin. Lima, Peru: International Potato Center (CIP).

[fsn3574-bib-0014] Hoover, R. (2001). Composition, molecular structure, and physicochemical properties of tuber and root starches: A Review. Carbohydrate Polymers, 45, 253–267. https://doi.org/10.1016/S0144-8617(00)00260-5

[fsn3574-bib-0015] Ikegwu, O. J. , Nwobasi, V. N. , Odoli, M. O. , & Oledinma, N. U. (2009). Evaluation of the pasting and some functional properties of starch isolated from some improved cassava varieties in Nigeria. African Journal of Biotech, 8(10), 2310–2315.

[fsn3574-bib-0016] Leach, H. W. , McCowen, L. D. , & Scotch, T. J. (1959). Structure of starch granule 1. Swelling and solubility patterns of various starches. Cereal Chemistry, 36, 534–544.

[fsn3574-bib-0017] McGill, A. S. , Hard, R. , Burt, J. R. , & Gunstone, F. D. (1974). Hept‐cis‐4‐enal and its contribution to the off‐flavor in cold stored cod. Journal of the Science of Food and Agriculture, 25, 1477–1489. https://doi.org/10.1002/(ISSN)1097-0010

[fsn3574-bib-0018] Melo, E. A. , Silva, M. P. , Krieger, N. , & Stamford, T. L. M . (2003). Physicohemical properties of yam bean (*Pachyrhizus erosus*. L. Urban) starch. Starch, 46, 245–247. 10.1016/s0960-8524(02)00313-9 12676508

[fsn3574-bib-0019] Moorthy, S. N. , & Ramanujam, T. (1986). Variation in properties of starch in cassava varieties in relation to age of the crop. Starch Starke, 38, 58–61. https://doi.org/10.1002/(ISSN)1521-379X

[fsn3574-bib-0020] Norman, A. S. M. , Hoque, M. A. , Haque, M. M. , Pervin, F. , & Karim, M. R. (2007). Nutritional and anti‐nutritional components in *Pachyrhyzus erosus* L. tuber. Food Chemistry, 102, 1112–1118. https://doi.org/10.1016/j.foodchem.2006.06.055

[fsn3574-bib-0021] Oguntunde, A. O. (1987). Review: Starch modification for food application. Nigerian Food Journal, 5, 102–107.

[fsn3574-bib-0022] Osundahunsi, O. F. , Fagbemi, T. N. , Kesselman, E. , & Shimoni, E. (2003). Comparison of the physicochemical properties and pasting characteristics of flour and starch from red and white sweet potato cultivars. Journal of Agricultural Food Chemistry, 51, 2232–2236. https://doi.org/10.1021/jf0260139 12670162 10.1021/jf0260139

[fsn3574-bib-0023] Padonou, W. , Mestres, C. , & Coffinago, M. (2005). The quality of boiled cassava roots: Instrumental characterization and relationship with physicochemical properties and sensorial properties. Food Chemistry, 89, 261–270. https://doi.org/10.1016/j.foodchem.2004.02.033

[fsn3574-bib-0024] Pearson, D. (1970). The Chemical Analysis of Foods, 6th ed. London: J & A Churchill.

[fsn3574-bib-0025] Plahar, W. A. , Annan, N. T. , & Nti, C. A. (1997). Cultivar and processing effects on pasting characteristics, tannin content and protein quality and digestibility of cowpea (*Vignaunguiculata*). Plant Foods for Human Nutrition, 51, 343–356. https://doi.org/10.1023/A:1007994612607 9650727 10.1023/a:1007994612607

[fsn3574-bib-0026] Richard, J. E. , Asaoka, M. , & Blanshard, M. V. (1991). The physicochemical properties of cassava starch. Tropical Science, 31, 189–207.

[fsn3574-bib-0027] Sabanis, D. , & Tzia, C. (2009). Effect of rice, corn and soy flour addition on characteristics of bread produced from different wheat cultivars. Food Bioprocess Technology, 2, 68–79. https://doi.org/10.1007/s11947-007-0037-7

[fsn3574-bib-0028] Sanni, L. O. , Ikuomola, D. P. , Sanni, S. A . (2001). Effect of length of fermentation and varieties on the qualities of sweet potato gari. Proc.8th triennial Symposium of the International Society for Tropical Root Crops. Africa Branch (ISTRC‐AB), Ed. M.O. Akoroda, IITA, Ibadan, Nigeria, 12‐16 November 2001, pp. 208‐211.

[fsn3574-bib-0029] Shimelis, E. , Meaza, M. , & Rakshit, S. (2006). Physicochemical properties, pasting behaviour and functional characteristics of flour and starches from improved bean (*Phaseolus vulgaris* L.) varieties grown in East Africa. East African Journal of Agricultural Engineering International, 8, 1–18.

[fsn3574-bib-0030] Shuey, W. C. , & Tipples, K. H . (1982). The Amylograph Handbook. St. Paul, MN: American Association of Cereal Chemists.

[fsn3574-bib-0031] Simsek, S . (2015). Wheat quality and carbohydrate research. Flour Analysis. http://www.ndsu.edu/faculty/simsek/wheat/flour.html# [last accessed: 11 October 2017]

[fsn3574-bib-0032] Singh, N. , Singh, J. , Kaur, L. , Sodhi, N. S. , & Gill, B. S. (2003). Morphological, thermal and rheological properties of starches from different botanical sources. Food Chemistry, 81, 219–231. https://doi.org/10.1016/S0308-8146(02)00416-8

[fsn3574-bib-0033] Sørensen, M. (1988). A taxonomic revision of the genus *Pachyrhizus* . DC. Nordic Journal of Botany, 8(2), 167–192. https://doi.org/10.1111/j.1756-1051.1988.tb00499.x

[fsn3574-bib-0034] Swinkles, J. M. (1985). Composition and properties of commercial nature starches. Starch Starke, 37(1), 1–5. https://doi.org/10.1002/(ISSN)1521-379X

[fsn3574-bib-0035] Ukpabi, J. U. (2010). Farmstead bread making potential of lesser yam (*Discorea esculenta*) flour in Nigeria. Australian Journal of Crop Science, 4(2), 68–72.

[fsn3574-bib-0037] Wooton, M. , & Bamunuarachahi, A. (1978). Water‐binding capacity of commercially‐ produced native and modified starches. Starches/Staerke, 30, 306–309. https://doi.org/10.1002/(ISSN)1521-379X

[fsn3574-bib-0038] Zanklan, A. S. , Ahouangonou, S. , Becker, H. C. , Pawelzik, E. , & Grüneberg, W. (2007). Evaluation of the storage root–forming legume yam bean (*Pachyrhizus spp*.) under West African conditions. Crop Science, 47, 1–14.

